# Fabrication of Isolated Iron Nanowires

**DOI:** 10.1021/acs.jpclett.3c02362

**Published:** 2023-09-18

**Authors:** David C. Grinter, Bobbie-Jean A. Shaw, Chi L. Pang, Chi-Ming Yim, Christopher A. Muryn, Charlotte A. Hall, Francesco Maccherozzi, Sarnjeet S. Dhesi, Masahiko Suzuki, Tsuneo Yasue, Takanori Koshikawa, Geoff Thornton

**Affiliations:** † Department of Chemistry and London Centre for Nanotechnology, University College London, London, WC1H 0AJ, U.K.; ‡ Diamond Light Source Ltd, Diamond House, Harwell Science and Innovation Campus, Didcot, OX11 0DE, U.K.; § School of Chemistry, University of Manchester, Manchester, M13 9PL, U.K.; ∥ Department of Chemistry, University of Reading, Reading, RG6 6AD, U.K.; ⊥ Fundamental Electronics Research Institute, Osaka Electro-Communication University, Neyagawa-shi, Osaka 572-8530, Japan

## Abstract

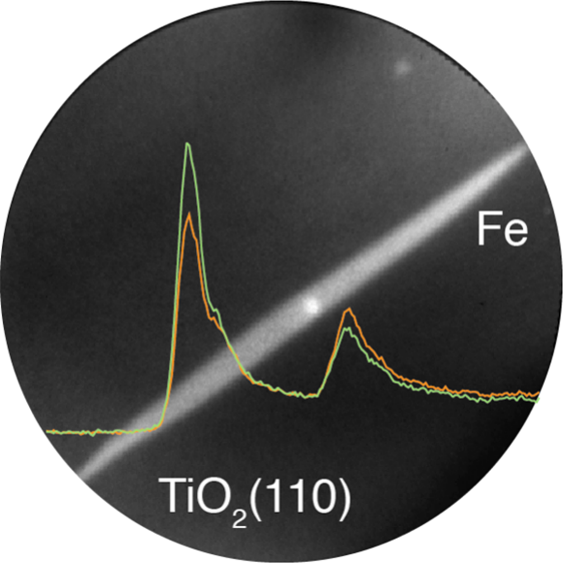

Nanoscale interconnects are an important component of molecular electronics. Here we use X-ray spectromicroscopy techniques as well as scanning probe methods to explore the self-assembled growth of insulated iron nanowires as a potential means of supplying an earth abundant solution. The intrinsic anisotropy of a TiO_2_(110) substrate directs the growth of micron length iron wires at elevated temperatures, with a strong metal–support interaction giving rise to ilmenite (FeTiO_3_) encapsulation. Iron nanoparticles that decorate the nanowires display magnetic properties that suggest other possible applications.

The potential of single molecule transistors to further the miniaturization of electronics remains an attractive goal.^1^ A key challenge lies in the fabrication of interconnects, with self-assembled nanostructures showing considerable promise.^2,3^ In this work we make use of the remarkable properties of TiO_2_ to construct oriented encapsulated metallic wires of nanometer dimensions. The surface properties of TiO_2_ have been studied extensively for more than five decades following the discovery of its photocatalytic properties.^4,5^ Since then tremendous progress has been made in this field, and the applications of TiO_2_ have expanded into a variety of technological areas including gas sensing, heterogeneous catalysis, corrosion protection, and electrical devices.^6^

Metal nanoparticles on metal oxide supports have been studied extensively due to their wide-ranging technological applications. This is especially the case for rutile TiO_2_(110), which is the prototypical metal-oxide surface for fundamental research. Moreover, the TiO_2_(110)-(1 × 1) surface is anisotropic (see Figure S1), which facilitates the directed growth of nanostructures,^7,8^ and the 3 eV band gap ensures electrical isolation of the conducting nanostructures from the substrate. Iron wires are investigated here, as the element is earth abundant and the wires offer potential in magnetic applications. As well as promoting self-assembly of metallic wires, the TiO_2_(110) substrate is also known to encapsulate metal nanostructures with oxides at elevated temperatures.^9,10^ This so-called strong metal support interaction (SMSI)^8,10−12^ provides a potential means to insulate the metallic wires.

In this Letter, we investigate the magnetic, chemical, and topographic properties of Fe nanowires grown on rutile TiO_2_(110)(1 × 1) using X-ray spectromicroscopy techniques and scanning probe methods. The results suggest a fabrication strategy for insulated metal nanowires with potentially useful magnetic properties.

Scanning tunneling microscopy (STM) in London was used to determine the optimum growth conditions for the Fe nanowires. X-ray photoemission electron microscopy (XPEEM) and spin-polarized low energy electron microscopy (SPLEEM) experiments were conducted on the I06 beamline at Diamond Light Source^13^ and at Osaka Electro-Communication University,^14^ respectively (see the Experimental Methods in the Supporting Information). Rutile TiO_2_(110) crystals were prepared via multiple cycles of argon ion sputtering and annealing in UHV (∼1000 K) until a sharp (1 × 1) low energy electron diffraction (LEED) pattern was obtained and contamination was below the detection level of Auger electron spectroscopy (AES). Fe metal was deposited via physical vapor deposition in UHV from an electron-beam evaporator, while the TiO_2_(110) crystal was held at an elevated temperature (∼1070 K). LEED and AES results from Fe/TiO_2_(110) are shown in Figure S2.

The deposition of Fe at elevated temperatures results in the formation of two types of nanostructures, namely, nanowires oriented along the [001] direction of the substrate (height ∼1 nm) and flat-topped pseudohexagonal islands (height ∼8 nm), as seen in Figure 1A and Figure 1B, respectively. This is a similar behavior to that observed for Pd/TiO_2_(110),^9^ with the size and morphology of the resulting structures being tuned by variations to the substrate temperature and deposition amount. The elongation of the nanowires along the [001] direction is driven by the strain^15^ induced by the anisotropy of the TiO_2_(110) substrate. This gives rise to a lattice mismatch between the substrate and an Fe(110) (bcc) overlayer of about 3% in the [001] direction and 12% in the [11̅0] direction.^16^ Also visible in Figure 1A are regions of reconstructed TiO_2_(110)-(1 × 2), formed as the surface becomes oxygen-deficient during the high-temperature deposition of Fe.

**Figure 1:**
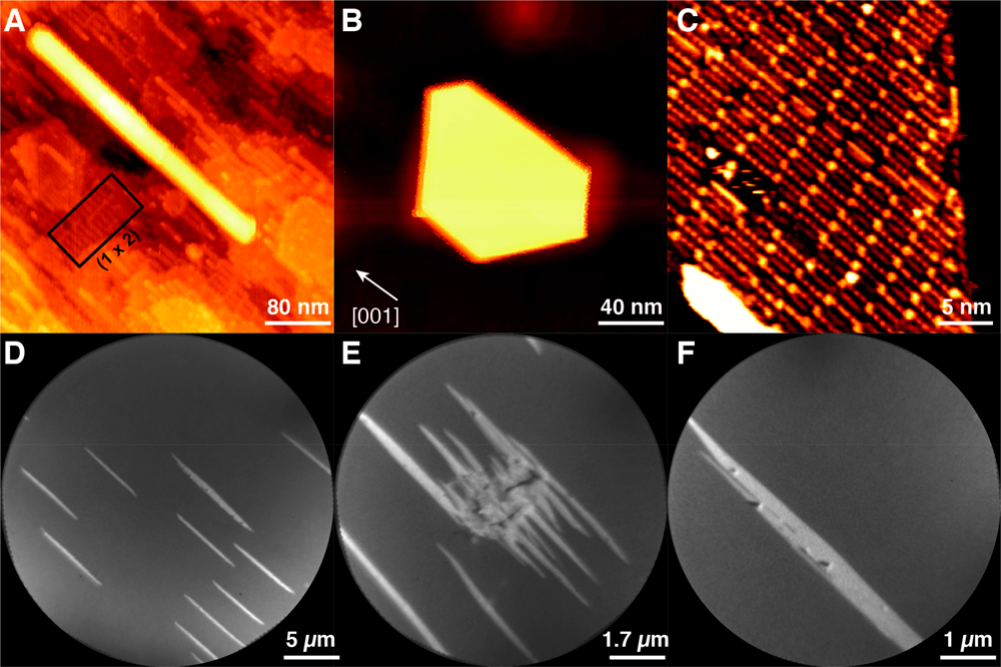
Structural characterization of iron nanostructures prepared on TiO_2_(110) at 1070 K. (A) STM image of an Fe nanowire (*V*_s_ = +1.0 V, *I*_t_ = 0.2 nA) recorded after deposition of 1 MLE Fe. (B) STM image of a pseudohexagonal Fe nanoisland (*V*_s_ = +2.9 V, *I*_t_ = 0.07 nA) recorded after deposition of 1 MLE Fe. (C) High-resolution image of the top surface of the Fe islands (*V*_s_ = +3.0 V, *I*_t_ = 0.14 nA). (D–F) XPEEM images of Fe nanowires recorded after deposition of 10 MLE Fe at 1070 K (*h*ν = 708 eV, KE = 4 eV). All images have the same orientation with respect to the TiO_2_(110) substrate.

An atomically resolved image of the surface of the pseudohexagonal island in Figure 1B is displayed in Figure 1C. The surface is composed of regular parallel rows of bright atomic-scale features aligned in the [001] direction of TiO_2_(110) and parallel to the long growth direction of the nanowires and resembles a modified Fe(110)-O “A” surface formed by O_2_ adsorption on Fe(110), as described by Freindl et al.^17^ The atomic-scale surface structure of the nanowires was observed in STM to be identical to that of the pseudohexagonal islands (see Figure S3). The presence of O on the surface of the nanostructures is expected due to facile migration/spillover of oxygen from the TiO_2_(110) substrate promoted by the elevated temperature during deposition, a clear indication of a strong metal support interaction (SMSI).^18^

To grow wires in preference to pseudohexagonal islands, a greater amount of iron was deposited than in the STM experiment (∼10 monolayer equivalents (MLE) vs ∼1 MLE). This also had the side effect of a 10× longer time period at high temperature (the dosing rate was the same), which promoted encapsulation with a metal oxide. Figure 1D–F shows Fe L_3_-edge XPEEM images (*h*ν = 708 eV) of nanowires deposited onto TiO_2_(110) at ∼1070 K. The images show the presence of several Fe-containing nanowires with lengths of 5–10 μm and widths up to ∼500 nm. Postanalysis with atomic force microscopy (AFM) showed that these nanowires had heights of <20 nm (Figure S4). The secondary-electron XPEEM measurements collect electrons with kinetic energies lower than 4 eV and as such will give a sampling depth in the range 5–10 nm,^19^ so that the core of the nanowires will be sampled. The average height of the nanowires was about 14 nm. Small dot-like features decorate the surface of some of the nanowires as seen in Figure 1D and F. Additionally, large micrometer-sized irregularly shaped clusters were occasionally observed on the surface, an example of which is displayed in Figure 1E, which acted as a nucleation point for several nanowires. This feature was identified through X-ray absorption spectroscopy (XAS) and X-ray photoelectron spectroscopy (XPS) as calcium, a common bulk contaminant of rutile TiO_2_(110) samples.^5^ However, Ca was absent from most of the nanowires investigated.

X-ray absorption spectroscopy (XAS) at the Ti L edge (Figure 2) is used to compare the titanium species of the TiO_2_(110) substrate (A) and within the encapsulation nanowires (B). The spectrum of the substrate matches that expected from the TiO_2_(110) literature;^20^ however, the spectrum obtained from the nanowires is rather different: in particular, we note the lack of splitting of the e_g_ band at the L_3_ edge and the differing intensity at the L_2_ edge (substrate, t_2g_ band > e_g_ band; wires, e_g_ band > t_2g_ band). The spectrum of the encapsulation layer around the nanowires matches very well with those reported in the literature for ilmenite (FeTiO_3_).^21−23^ Ordered ilmenite structures have been previously reported for low coverages of iron deposited onto the TiO_2_(011) surface under slightly oxidizing conditions, although these proved to be unstable at high annealing temperatures in contrast to the encapsulation layers here.^24^

**Figure 2:**
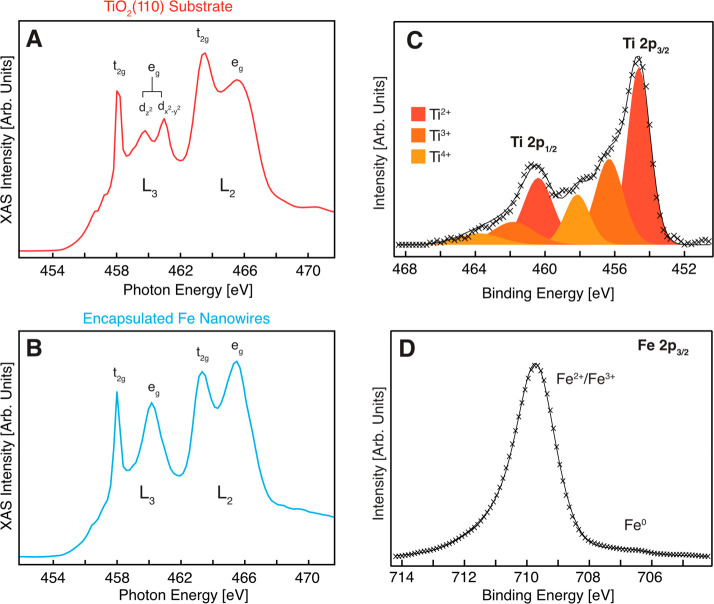
The nature of the Ti species on the encapsulated nanowires and oxidation states of Ti and Fe associated with the nanowires and substrate. Ti L-edge XAS spectra of the Fe/TiO_2_(110) system acquired from XPEEM images (KE = 4 eV), with sampling of areas corresponding to the bare substrate (A, red curve) and the Fe nanowires (B, blue curve). (C) Ti 2p XPS spectrum (*hν* = 650 eV) and (D) Fe 2p XPS spectrum (*hν* = 820 eV), obtained from the Fe nanowires supported on TiO_2_(110) with bare substrate in-between.

Photoemission is used to probe the near surface of the nanowires, with Ti 2p (*hν* = 650 eV) and Fe 2p spectra (*hν* = 820 eV) probing to a depth of 4–5 nm.^25^ The Ti 2p XPS spectrum (obtained via the micro-XPS mode of the XPEEM instrument, which sampled the bare substrate as well as a number of nanowires) reveals the presence of three types of titanium species, namely, Ti^2+^, Ti^3+^, and Ti^4+^, as displayed in Figure 2C. A Shirley type background was subtracted from the data, and each Ti 2p doublet was fitted to three contributions with Voigt lineshapes (30:70 Gaussian–Lorentzian ratio), corresponding to Ti^2+^, Ti^3+^, and Ti^4+^ species. The fitting of these overlapping features requires the imposition of certain restraints such as the peak area and position; the area of the peaks in the Ti 2p_1/2_ region was constrained to half that of the Ti 2p_3/2_ region, the spin–orbit separation of each oxidized Ti species was held constant at 5.7 eV, and the Ti^2+^–Ti^3+^ and Ti^3+^–Ti^4+^ energy separation for each multiplet peak was set to 1.7 and 1.8 eV, respectively.^26^ The presence of reduced Ti species is partially indicative of photon-induced reduction of the TiO_2_, as has been reported previously from similar microfocused undulator beamlines,^27^ in addition to the thermally induced surface reduction during the Fe deposition process. As micro-XPS also sampled some of the nanowires, the reduced Ti species may also originate from the encapsulation layer. From XPEEM imaging we estimate that approximately 5% of the surface region sampled for the data in Figure 2 was covered by nanowires.

The XPS spectrum of the Fe 2p region of the nanowires (also obtained in the micro-XPS mode of the XPEEM instrument, which sampled a region containing a few nanowires as well as the bare substrate) is displayed in Figure 2D. The interpretation of Fe 2p XPS is challenging, especially in the case of mixed-oxide systems. Nevertheless, the low binding energy feature at 706.6 eV matches well with that reported for metallic Fe^0^, and the main peak at 709.7 eV is likely to be related to oxidized Fe^2+^ and Fe^3+^ species along with their complex multiplet structure.^28^ An Fe^2+^ contribution is expected for an ilmenite layer, with Fe^3+^ possibly arising from Fe_2_O_3_ at the interface with the iron nanowires. The faint metallic iron component (<5% of the total peak area) suggests that the encapsulation layer has a thickness of around 0.5 nm, given the probing depth at a photoelectron kinetic energy of ∼100 eV. Given the total wire thickness of ∼14 nm (see Figure S4) and assuming a uniform encapsulation layer, this reflects a metallic iron contribution of ∼85% of the total wire volume.

X-ray magnetic circular dichroism (XMCD) XPEEM measurements were used to probe the magnetic behavior of the iron nanowires. Figure 3A shows an XAS image obtained at the maximum of the Fe L_3_ edge (*h*ν = 708 eV). The nanowires (elongated stripes) and bright nanodots are clearly visible on the darker TiO_2_(110) substrate. An XMCD (magnetic contrast) image was recorded at the L_3_ edge and is displayed in Figure 3B. The XMCD image was calculated from two sets of images recorded at the Fe L_3_ peak, normalized with the off-resonance image, with right (μ_+_) and left (μ_–_) circularly polarized light, as (μ_+_ – μ_*-*_)/(μ_–_ + μ_+_). Domains magnetized parallel or antiparallel to the polarization vector will appear black or white in the XMCD image, while domains with a magnetization perpendicular to the polarization vector will have a gray contrast (corresponding to zero XMCD asymmetry).^29^ In the Figure 3C data, there is evidence that the nanodots are well magnetized as they display a pronounced contrast in the XMCD image. The nanowires, however, do not display any sizable contrast in the XMCD images in Figure 3B. Further XMCD images of nanodots from other regions of the sample are displayed in Figure S5. Parts C and D of Figure 3 show an XMCD image and an XAS image, respectively, where the nanodots display opposite contrast in XMCD at the L_3_ edge (see line profiles in Figure 3E), indicating their opposite magnetization directions.

**Figure 3:**
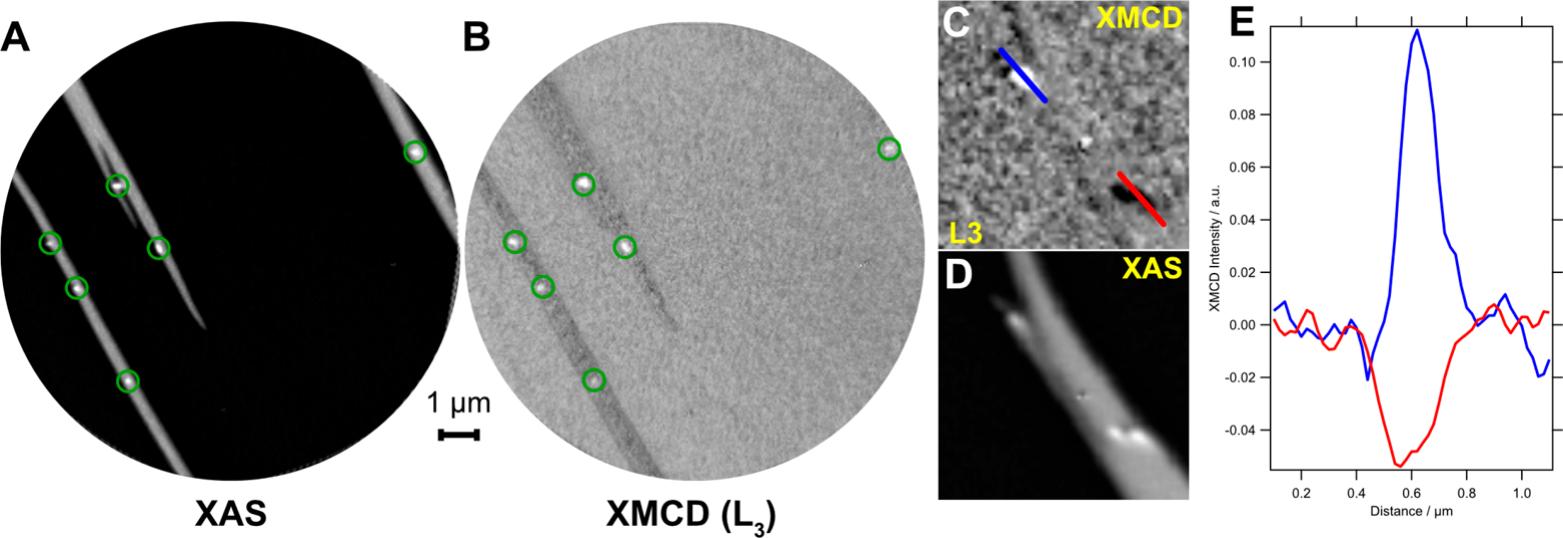
Magnetic behavior of the nanowires and dots. XMCD-XPEEM (KE = 4 eV) images of Fe nanowires and nanodots (green circles in parts A and B) supported on TiO_2_(110) at the Fe L_3_ edge (*h*ν = 708 eV). (A) XAS image and (B) XMCD-XPEEM image of the same 10 μm FOV. (C) XMCD image highlighting a few of the nanodots. (D) XAS image of the same area as part C, showing the morphology of the dots and wire (2 × 2 μm^2^). (E) Line profiles across two of the nanodots from the XMCD image in part C.

The Fe L_2,3_-edge X-ray absorption spectra acquired from the nanodots and the nanowires imaged in Figure 3A are displayed in Figure 4. Figure 4A shows the integrated XAS spectra (normalized to the pre-edge region) calculated by sampling stacks of XPEEM images to acquire spatially resolved XAS as well as right (μ_+_) and left (μ_–_) circularly polarized spectra. The green and orange curves were collected from the nanodots and the nanowires, respectively. As expected from the intensity of the two species observed in the image in Figure 3A, the dots display a higher overall intensity, as well as a slightly different line shape. The Fe L_2,3_-edge absorption spectra of the nanowires very closely match that of FeTiO_3_, primarily composed of octahedral Fe^2+^ with the main L_3_ peak at 708 eV accompanied by a shoulder characteristic of Fe^3+^ at 710 eV and the L_2_ peak at 720.8 eV;^21^ the results are consistent with the mixed-oxide view from our XPS data in Figure 2. The lower panel of Figure 4A displays the calculated (using CRISPY)^30^ XAS spectrum of Fe^2+^ in an octahedral geometry (pink dashed line) showing particularly good agreement with the fine structure of the L_2_ edge for the nanowires. This mixed oxide is expected due to the high temperature sample preparation as titanium oxides spill over onto the iron wires, forming the encapsulating ilmenite film, as previously seen for other metals on TiO_2_(110).^8−12^ The nanodots display a less pronounced L_3_ shoulder at 710 eV and a quite different L_2_ edge, where the feature at 719.4 eV is no longer present, indicating a lower amount of Fe^3+^ and more metallic character. Figure 4B shows separate XAS spectra from the nanodots acquired with right (blue) and left (red) circularly polarized light, as well as the XMCD difference between the two (black, dashed line) and the calculated (using CRISPY)^30^ XMCD for octahedral Fe^2+^ (dashed pink line). Figure 4C shows the same set of spectra as those acquired from the nanowires. There is a clear XMCD signal from the nanodots compared with a very minor signal from the nanowires, in line with the results seen in the imaging experiments in Figure 3. The nanodot XMCD signal matches quite well with that predicted for Fe^2+^ (the pink line in the lower panel of Figure 4B) and other reported FeTiO_3_ systems.^21−23^

**Figure 4:**
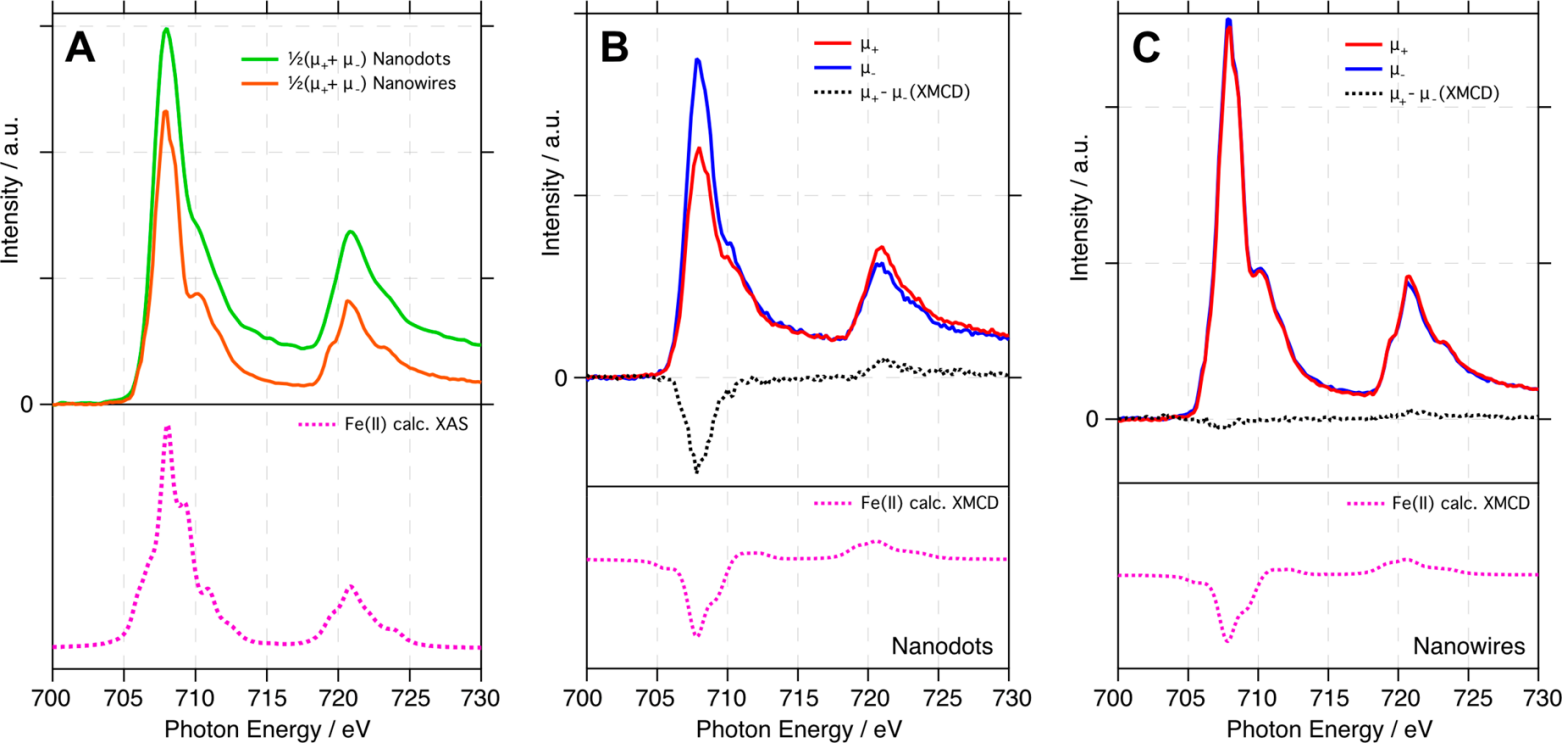
Fe L-edge XAS and XMCD spectra (KE = 4 eV) obtained from the Fe structures on TiO_2_(110). (A) Integrated XAS (average of right-circular and left-circular spectra) taken from regions of the images in Figure 3A corresponding to the nanodots (green) and nanowires (orange). Spectra are normalized to the pre-edge region. The calculated Fe^2+^ XAS spectrum is shown in the lower panel as a dashed pink line. (B) Circularly polarized XAS (blue, red lines) and XMCD (dashed black line) spectra from the nanodots. Spectra are normalized to the edge step. The lower panel shows the calculated XMCD spectrum for Fe^2+^ in octahedral geometry (dashed pink line). (C) Circularly polarized XAS (blue, red lines) and XMCD (dashed black line) spectra from the nanowires. Spectra are normalized to the edge step. The lower panel shows the calculated XMCD spectrum for Fe^2+^ in octahedral geometry (dashed pink line).

The system here contains a mixture of iron species as well as a nanosized object that has unknown band structures. Hence, rather than extracting the absolute values of the magnetic parameters *m*_*l*_ and *m*_*s*_ for the nanodots and wires from the spectra we use the ratio *m*_*l*_/*m*_*s*_, which only depends on the *p* and *q* values.^31,32^ The measurement of these from the XMCD spectra is shown in Figure S6 and Table ST1. We found the nanodots to have an *m*_*l*_/*m*_*s*_ ratio of 0.3 and the nanowires 0.22, quite far from the value for bulk iron (0.043)^33−36^ but close to values recorded for Fe_2_O_3_–FeTiO_3_ by Hojo et al. (*m*_*l*_/*m*_*s*_: 0.21 in plane, 0.14 out of plane; *H* = 10 T, *T* = 150 K)^23^ and similar to other Fe-containing systems.^31−33,36−40^ Overall, our XAS and XPS results present a complex picture that suggests that the nanowires and nanodots are composed of a metallic iron core with an encapsulation layer consisting of mixed Fe–Ti oxides, possibly a mixture of FeTiO_3_ and α-Fe_2_O_3_, which would explain the presence of the mixed valence state of Fe (Fe^0^, Fe^2+^, and Fe^3+^) and the existence of an XMCD signal. Moreover, the nanodots display a more metallic character, with a subsequent significantly higher XMCD signal.

In order to probe the magnetic behavior of the nanowires and nanodots in a more surface-sensitive manner, spin-polarized LEEM (SPLEEM) images were acquired on a different Fe/TiO_2_(110) sample prepared in the same way. At the starting voltages used for the images in Figure 5, the typical probe depth of the SPLEEM is around 0.4–0.5 nm. This compares with the secondary electron XPEEM results shown above, where the probe depth is up to a few nm. Despite the identical growth conditions, nanodots were not observed on these samples, and the only Fe-related structures formed were nanowires which displayed the same general morphology as those prepared for the synchrotron experiments. SPLEEM images of a typical wire are displayed in Figure 5, where parts A and B were acquired with the polarization vector of the incident beam (*P*_0_ = 90%) parallel to the [110] direction of the surface, along with the asymmetry image (Figure 5C). No magnetic contrast was observed in this asymmetry image. In order to examine possible orientation dependence, SPLEEM images were also acquired with *P* parallel to the [11̅0] and [001] crystallographic directions of the substrate, and the results also showed no magnetic contrast. The discrepancy of these results with the slight XMCD signal detected by the XMCD measurements shown in Figure 4 is due to the greater surface sensitivity of the electron-based probe compared to the soft X-rays, which do not probe the metallic iron in the core of the nanowires.

**Figure 5:**
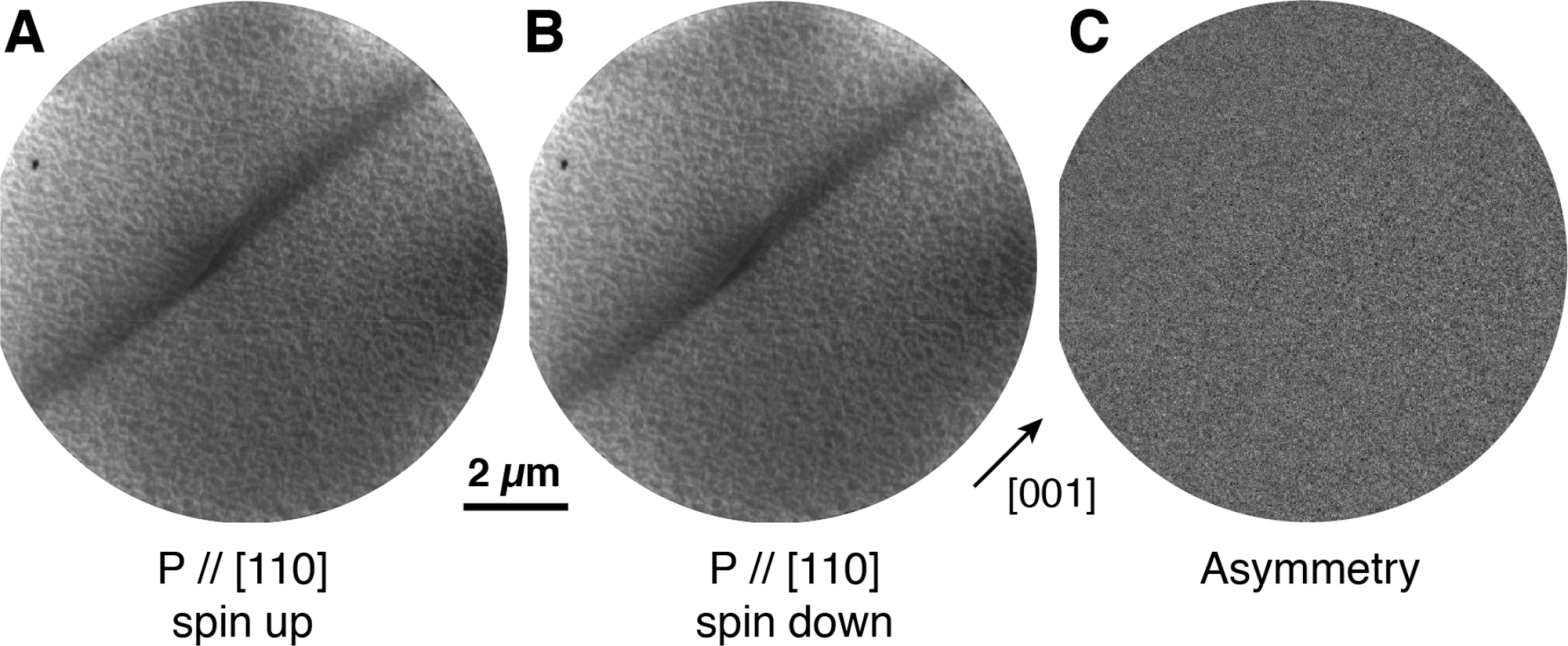
Spin-polarized LEEM images of a typical Fe nanowire on TiO_2_(110). The wire was prepared with the sample held at ∼1100 K. Parts A and B were acquired at room temperature with the electron beam polarization vector P // [110] with spin up and spin down, respectively. The resulting asymmetry image is displayed in part C. FOV = 10 μm, SV = 4.1 V.

In summary, insulated nanowires of metallic iron were grown on a rutile TiO_2_(110) support. These nanowires, insulated by encapsulating in a mixture of FeTiO_3_ and Fe_2_O_3_, are decorated with magnetic nanodots. This type of self-assembled wire, fabricated from earth-abundant materials, suggests its application as an interconnect in nanoscale electronics.

## Supporting Information Available

Additional details of the experimental and theoretical methods and further characterization of the nanowire system (AFM, STM, low energy electron diffraction, and XMCD spectro-microscopy) (PDF)
Transparent Peer Review report available (PDF)
