# Supplementary material for: Fabrication of Isolated Iron Nanowires
Source: J Phys Chem Lett. 2023 Sep 18;14(38):8507–12. doi: 10.1021/acs.jpclett.3c02362 (PMC10544030; doi:10.1021/acs.jpclett.3c02362)
Supplement: Supplementary file 1 — jz3c02362_si_001.pdf [file jz3c02362_si_001.pdf]

Fabrication of Isolated Iron Nanowires – Supporting Information

David C. Grinter[1,2], Bobbie-Jean A. Shaw,[1] Chi L. Pang[1], Chi-Ming Yim[1], Christopher A. Muryn[3], Charlotte A. Hall[2,4], Francesco Maccherozzi[2], Sarnjeet S. Dhesi[2], Masahiko Suzuki[5], Tsuneo Yasue[5], Takanori Koshikawa[5] and Geoff Thornton[1*]

[1]Department of Chemistry and London Centre for Nanotechnology, University College London, London, WC1H 0AJ, UK

[2]Diamond Light Source Ltd, Diamond House, Harwell Science and Innovation Campus, Didcot, OX11 0DE, UK

[3]School of Chemistry, University of Manchester, Manchester, M13 9PL, UK

[4]Department of Chemistry, University of Reading, Reading, RG6 6AD, UK

[5]Fundamental Electronics Research Institute, Osaka Electro-Communication University, Neyagawa-shi, Osaka 572-8530, Japan

**Corresponding Author: g.thornton@ucl.ac.uk*

Table of Contents

1. Experimental Methods
Figure S1. Structural model of $TiO_2(110)$
Figure S2. Characterisation with LEED/AES
Figure S3. STM images of encapsulation
Figure S4. AFM topography of nanowires
Figure S5. XMCD imaging
Figure S6. XMCD spectra
Table ST1. XMCD parameters

1. Experimental Methods

STM measurements were carried out at UCL using an Omicron UHV variable temperature STM, operated at 300 K. Synchrotron radiation studies were conducted on the I06 beamline at Diamond Light Source, using an Elmitec Spectroscopic Photoemission and Low Energy Electron Microscope (SPELEEM) capable of imaging with magnetic, topographic and chemical contrast, using low energy electrons or soft X-rays as a probe. This versatile instrument is able to perform an array of techniques, including X-ray Absorption Spectroscopy (XAS), micro X-ray Photoelectron Spectroscopy (μ-XPS), Low Energy Electron Diffraction (LEED), Low Energy Electron Microscopy (LEEM) and X-ray Photoemission Electron Microscopy (XPEEM). The insertion device at I06 is capable of delivering variable polarisations which permits X-ray Magnetic Circular Dichroism (XMCD) measurements. Post-experiment AFM characterization of the samples was performed using a Veeco CP-II AFM. The magnetic imaging experiments were carried out using a high-brightness and highly spin-polarised LEEM (*Elmitec* SPLEEM) located at the Osaka Electro-Communication University, Japan. SPLEEM images were acquired with orthogonal spin-polarization directions along the [110], [$1\bar{1}0$], and [001] crystallographic orientations of the substrate.

The temperatures during sample preparation and metal deposition were measured with an optical pyrometer (Minolta) and a type C thermocouple attached to the sample holder. The iron coverage is given in monolayer equivalents (MLE) where 1 MLE corresponds to the amount of iron required to form a complete monolayer across the surface. The Fe evaporator was calibrated using the well-known growth of Fe on W(110) to determine the time/flux required to deposit an individual monolayer. The photon energy was calibrated to the Ti L edge of the clean TiO_2(110) substrate and is estimated to have an error of ±0.2 eV at the Fe L edge. The XPS binding energies were calibrated to adventitious carbon at 284.8 eV.

The topography of the Fe nanowires was observed using STM, LEEM and AFM prior to chemical and magnetic characterisation with XPEEM, XAS and XPS. The XPS spectra were acquired using photon energies of 820 eV and 650 eV for Fe *2p* and Ti *2p*, respectively; O *1s* spectra were acquired using both of the aforementioned photon energies. Binding energies were calibrated to the adventitious C *1s* peak at 284.8 eV. XMCD spectra and images were acquired by calculating difference spectra (images) at the Fe $L_{2,3}$ XAS edge when recorded with opposing photon helicities. The azimuthal angle of incidence of the X-rays was varied through 180° to determine the optimal angle (i.e. where the magnetization direction of the sample is parallel to the light helicity).

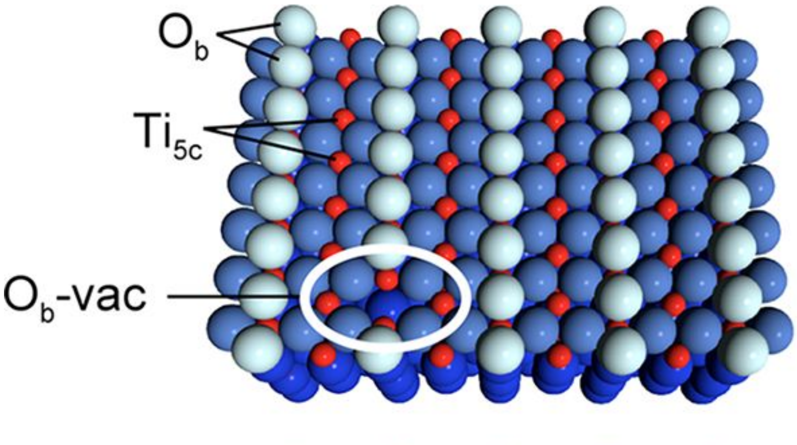

Figure S1. Model of the $TiO_2(110)$ surface. Blue and red spheres are lattice O and Ti, respectively. The light blue and red spheres are O_b and Ti_{5c} atoms, respectively. In UHV, the reduced surface has approximately 5% ML of oxygen vacancies (O_b-vac).

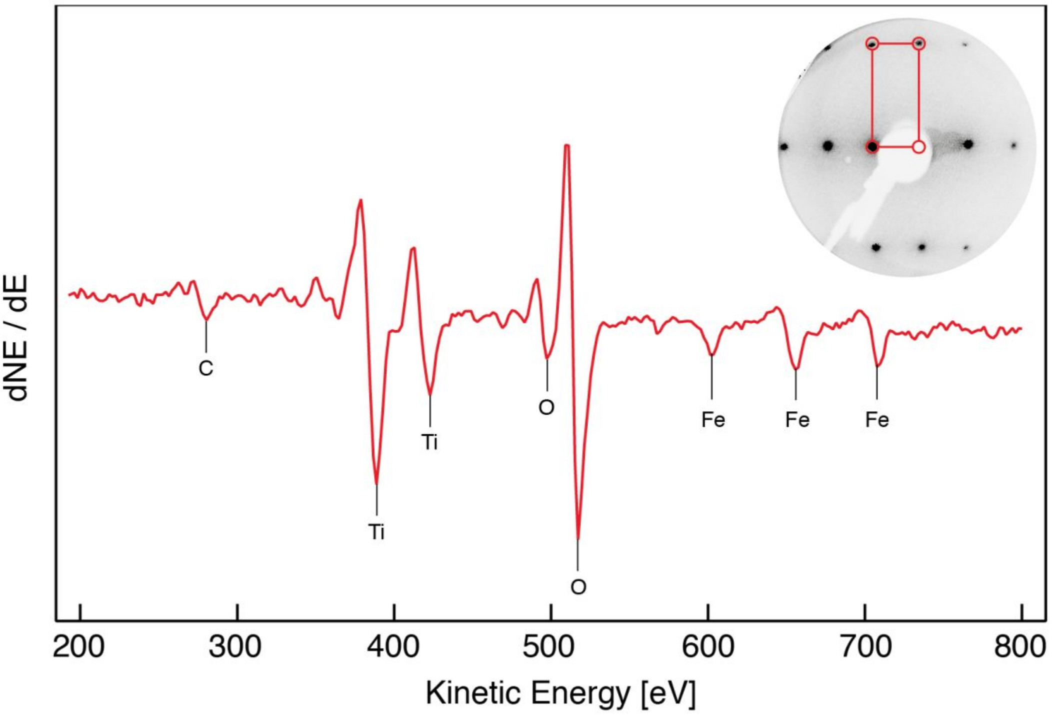

Figure S2. Auger electron spectrum of the Fe/TiO_2(110) system prior to the XPEEM measurements. (coverage ~ 9 ML) Inset is a low energy electron diffraction pattern of the clean TiO_2(110) substrate.

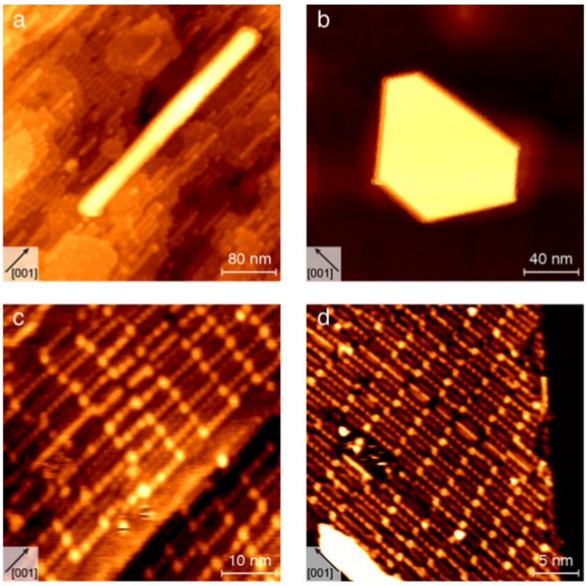

Figure S3. STM images of the top surfaces of an iron nanowire **(A, C)** and hexagonal island **(B, D)** on TiO_2(110).

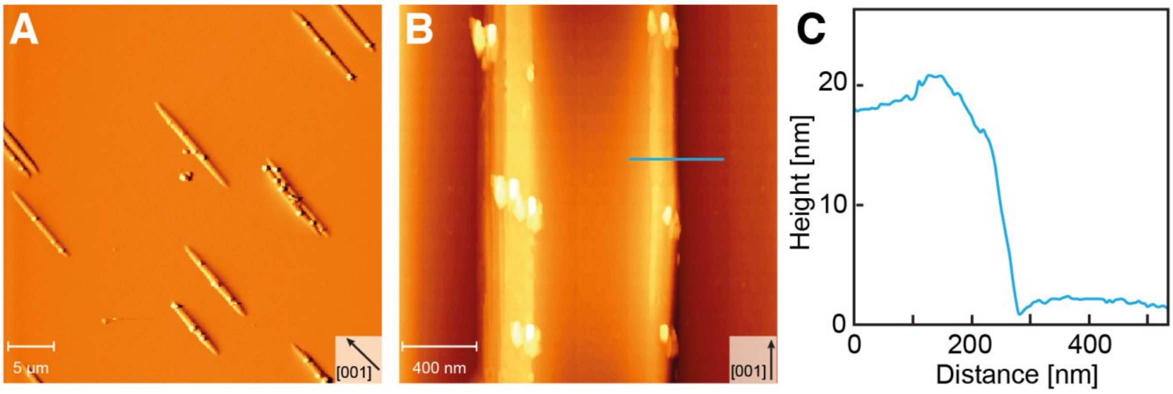

Figure S4. (A, B) Post-XPEEM experiment NC-AFM images of the Fe nanowires on $TiO_2(110)$ with associated line profile **(C)**. Precise measurement of the "nanodot" height is challenging due to tip-convolution effects.

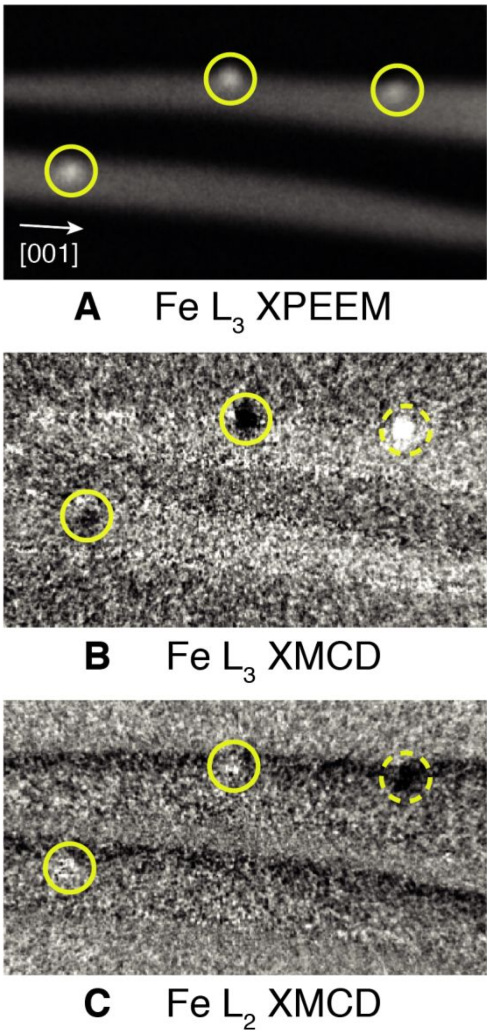

Figure S5. XMCD-XPEEM images ($1.7 \times 1\ \mu m^2$) of Fe nanowires and nanodots (highlighted) supported on $TiO_2(110)$. **A:** XPEEM image at the Fe L_3 edge, **B:** XMCD image of the same area at the Fe L_3 edge, **C:** XMCD image of the same area at the Fe L_2 edge.

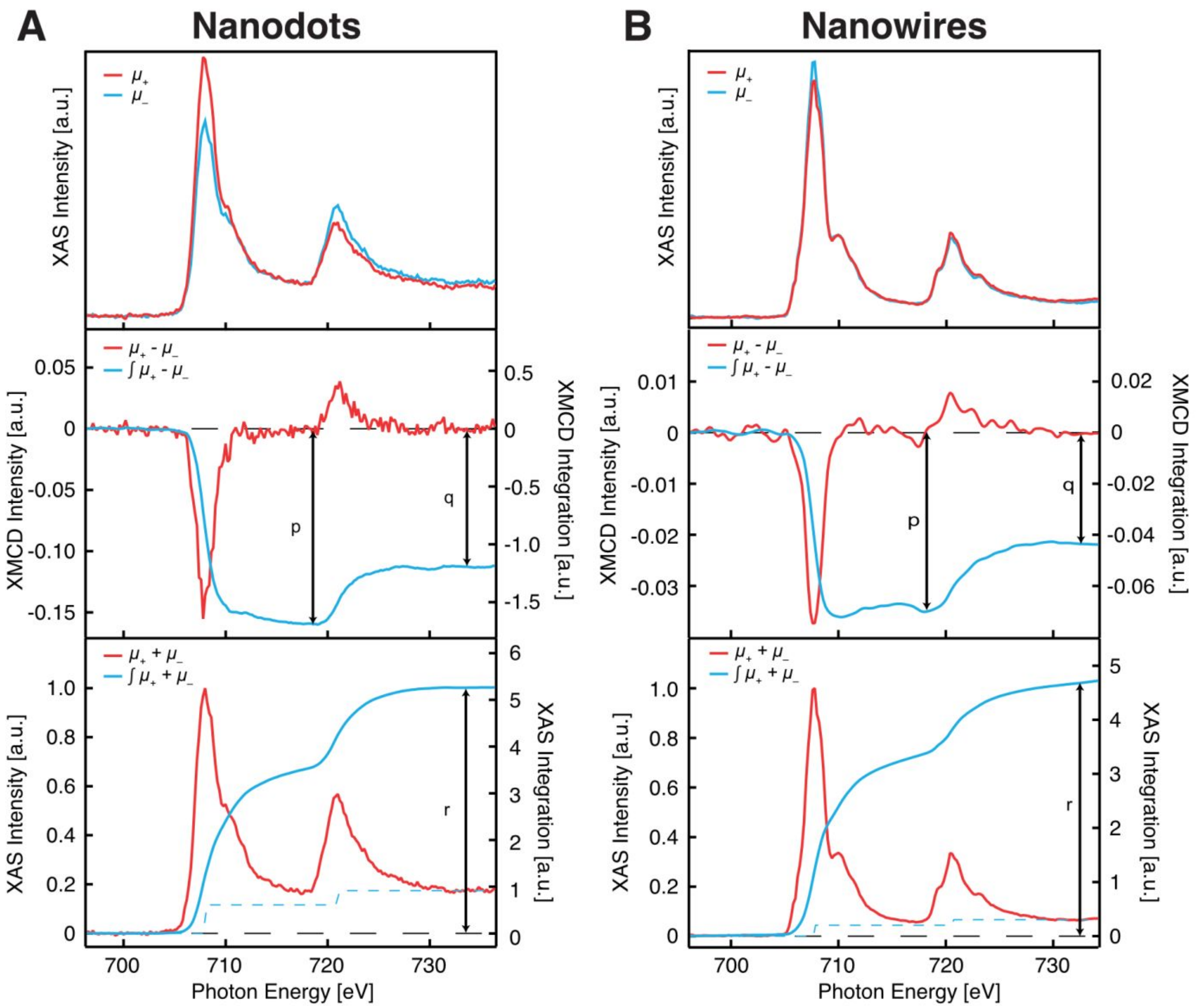

Figure S6. Fe L-edge XAS and XMCD spectra obtained from the Fe nanodots, (**A**) and nanowires, (**B**) respectively. The top plots show the XAS spectra obtained from XPEEM image stacks acquired with right and left circularly polarised light. The middle plots show the XMCD spectra resulting from the XAS difference spectra (red) and integration spectrum of the XMCD signal (blue). The bottom plots show the sum of the XAS spectra (red) and corresponding integration spectrum (blue) performed after a step-like background was removed (blue dashed line).

	**Nanodots**	**Nanowires**
**p**	1.80	0.072
**q**	1.28	0.043
**r**	5.08	4.72

Table ST1. Table of values measured from Figure S6.
