# Supplementary material for: Fabrication of Isolated Iron Nanowires
Source: J Phys Chem Lett. 2023 Sep 18;14(38):8507–12. doi: 10.1021/acs.jpclett.3c02362 (PMC10544030; doi:10.1021/acs.jpclett.3c02362)
Supplement: Supplementary file 2 — jz3c02362_si_002.pdf [file jz3c02362_si_002.pdf]

jz-2023-02362a.R1

Name: Peer Review Information for "Fabrication of Isolated Iron Nanowires"

First Round of Reviewer Comments

Reviewer: 1

Comments to the Author

Referee Report on submission to JPC Letters

by Thornton et al.

This is an excellent paper on an important topic: the self assembly of oriented, insulated metal nanowires. It is shown that insulated nanowires of metallic iron can be grown by simple vapor deposition of iron onto a rutile $TiO_2(110)$ surface at high temperature. The wires are all oriented in the same direction. These nanowires and shown to be insulated by encapsulating in a mixture of $FeTiO_3$ and Fe_2O_3, and decorated with magnetic nano-dots. This type of self-assembled wire may have application in nanoscale devices of several types.

The experimental evidence in support of these structural conclusions in excellent. It includes very impressive measurements with a range of X-ray spectromicroscopy techniques and scanning probe methods. Few teams in the world could have put together such an impressive and elegant set of analyses that are analyzed in such rigor and with such well-versed reference to the relevant prior literature. I congratulate the authors for this tour de force!

I only have minor suggestions for improvement:

1. The 2nd sentence reads: “This is especially the case for rutile $TiO_2(110)$, which is the prototypical metal-oxide surface.” I suggest to qualify this by adding the following phrase to the end: “for fundamental research”.

2. The elemental Fe signal in XPS is so weak (due to the short mean-free-path of these photoelectrons) that I find myself wishing that there were more proof that the dominant material

in the nanowires is metallic Fe and not some Fe oxide. Is there some way to prove this? (e.g., XPS using a much higher photon energy?) If so, it should be added. Or at least, use the weak signal in XPS, the probe depth and the nanowire thickness to estimate what fraction of the Fe in the wires is metallic Fe.

3. A sentence on p. 10 reads: “This mixed oxide is expected due to the high temperature sample preparation as titanium oxides spillover onto the iron wires, forming the encapsulating ilmenite film.” This expectation is surely based on some prior literature that should be cited here.

Author's Response to Peer Review Comments:

Response to Referee

This is an excellent paper on an important topic: the self assembly of oriented, insulated metal nanowires. It is shown that insulated nanowires of metallic iron can be grown by simple vapor deposition of iron onto a rutile TiO2(110) surface at high temperature. The wires are all oriented in the same direction. These nanowires and shown to be insulated by encapsulating in a mixture of FeTiO3 and Fe2O3, and decorated with magnetic nanodots. This type of self- assembled wire may have application in nanoscale devices of several types.

The experimental evidence in support of these structural conclusions in excellent. It includes very impressive measurements with a range of X-ray spectromicroscopy techniques and scanning probe methods. Few teams in the world could have put together such an impressive and elegant set of analyses that are analyzed in such rigor and with such well-versed reference to the relevant prior literature. I congratulate the authors for this tour de force!

I only have minor suggestions for improvement:

1. The 2nd sentence reads: “This is especially the case for rutile TiO2(110), which is the prototypical metaloxide surface.” I suggest to qualify this by adding the following phrase to the end: “for fundamental research”. 2. The elemental Fe signal in XPS is so weak (due to the short mean-free-path of these photoelectrons) that I find myself wishing that there were more proof that the dominant material in the nanowires is metallic Fe and not some Fe oxide. Is there some way to prove this? (e.g., XPS using a much higher photon energy?) If so, it should be added. Or at least, use the weak signal in XPS, the probe depth and the nanowire thickness to estimate what fraction of the Fe in the wires is metallic Fe.

3. A sentence on p. 10 reads: “This mixed oxide is expected due to the high temperature sample preparation as titanium oxides spillover onto the iron wires, forming the encapsulating ilmenite film.” This expectation is surely based on some prior literature that should be cited here.

We thank the referee and editor for their time and useful comments and have addressed the issues a point-by point basis below:

1. We have made the suggested addition of "for fundamental research" on page 2.

2. Unfortunately, we only have XPS of this system at the single photon energy presented, so a precise estimate of the thickness of the iron core is challenging. We have added the following sentence to page 7 to clarify our estimate.

   "The faint metallic iron component (< 5% of the total peak area) suggests that the encapsulation layer has a thickness of around 0.5 nm, given the probing depth at a photoelectron kinetic energy of ~100 eV. Given the total wire thickness of ~14 nm (see Figure S4), and assuming a uniform encapsulation layer, this reflects a metallic iron contribution of ~85 % of the total wire volume."

3. We have added references (8-12) to this sentence on page 10 which illustrate similar encapsulation of metal adsorbates on TiO_2(110) at high temperature.
